# Divide or Commit – Revisiting the Role of Cell Cycle Regulators in Adult Hippocampal Neurogenesis

**DOI:** 10.3389/fcell.2019.00055

**Published:** 2019-04-24

**Authors:** Anja Urbach, Otto W. Witte

**Affiliations:** Hans Berger Department of Neurology, Jena University Hospital, Jena, Germany

**Keywords:** dentate gyrus, neural stem cells, cyclins, cyclin-dependent kinases, proliferation, differentiation, fate determination, G_1_

## Abstract

The adult dentate gyrus continuously generates new neurons that endow the brain with increased plasticity, helping to cope with changing environmental and cognitive demands. The process leading to the birth of new neurons spans several precursor stages and is the result of a coordinated series of fate decisions, which are tightly controlled by extrinsic signals. Many of these signals act through modulation of cell cycle (CC) components, not only to drive proliferation, but also for linage commitment and differentiation. In this review, we provide a comprehensive overview on key CC components and regulators, with emphasis on G_1_ phase, and analyze their specific functions in precursor cells of the adult hippocampus. We explore their role for balancing quiescence versus self-renewal, which is essential to maintain a lifelong pool of neural stem cells while producing new neurons “on demand.” Finally, we discuss available evidence and controversies on the impact of CC/G_1_ length on proliferation versus differentiation decisions.

## Introduction

**FIGURE 1:**
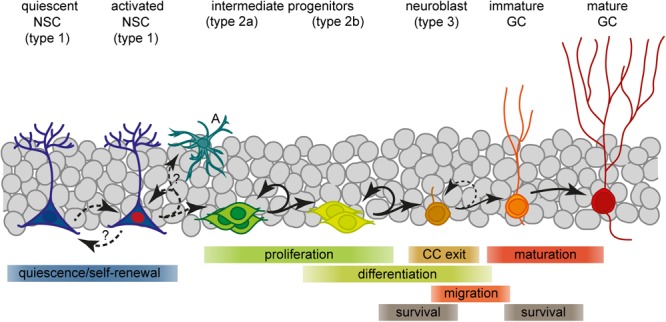
Scheme of the current view of lineage progression and fate decisions during adult hippocampal neurogenesis. A, astrocyte; GC, granule cell; NSC, neural stem cell.

The presence of NSC capable of generating new neurons throughout life provides adult mammals with an exceptional level of brain plasticity. aNSC are multipotent, have the capacity to self-renew and generate progenitor cells which give rise to neurons that functionally integrate into pre-existing networks (Zhao et al., 2006; Suh et al., 2007; Toni et al., 2008; Zhao et al., 2008; Bonaguidi et al., 2011; Encinas et al., 2011; Mongiat and Schinder, 2011; Pilz et al., 2018). Under physiological conditions, adult neurogenesis is mainly restricted to two brain regions: the SVZ of the lateral ventricles and the SGZ of the DG (Doetsch, 2003; Alvarez-Buylla and Lim, 2004). Persuasive evidence suggests that adult-born neurons participate in specific brain functions, including learning and memory, mood regulation, and pheromone-related behaviors (Ming and Song, 2011). Adult neurogenesis is a dynamic, finely tuned process that involves repeated fate decisions, including the proliferation and differentiation of stem and progenitor cells, or survival and maturation of newborn neurons (Figure 1). Sustaining the balance between such fate decisions is critical for maintaining homeostasis in the system, as excessive proliferation may induce stem cell exhaustion followed by premature depletion of their pool (Kippin et al., 2005; Sierra et al., 2015). On the other hand, disordered generation of new neurons might disturb the function of the circuit in which they integrate and contribute to neurological disorders (Zhao et al., 2008; Ruan et al., 2014; Jessberger and Parent, 2015). Hence, every step of adult neurogenesis must be tightly regulated by a complex interplay of extrinsic and intrinsic genetic factors to facilitate proper circuit adaption to changing environmental demands (Zhao et al., 2008; Ming and Song, 2011; Faigle and Song, 2013; Opendak and Gould, 2015). In a wide array of cellular contexts, including neural cells, cell fate decisions are closely linked to the CC. In particular the G_1_ phase opens a time window through which cells can respond to mitogens and specification signals to execute their decision to divide, differentiate or exit the CC (Blomen and Boonstra, 2007; Salomoni and Calegari, 2010; Boward et al., 2016).

We will herein discuss recent progress in our understanding of how the CC integrates extrinsic signals to regulate processes leading to the birth of a new neuron, with emphasis on the G_1_ phase. Since particularly the hippocampal newborn neurons play critical roles in learning and memory, with disturbances in their generation being associated to neurologic diseases and aging-related cognitive decline, we focus our review on the adult DG. Beyond that, we will discuss relevant findings obtained in the embryonic brain for which more comprehensive data exist, and draw comparisons to the SVZ, where applicable.

## Neurogenesis in the Adult Dentate Gyrus

Neural stem cells residing in the hippocampal SGZ, a narrow area between the dentate granule cell layer and the hilus, are a source of lifelong neurogenesis. They generate intermediate progenitors (IPCs) which give rise to neuroblasts that exit the CC and convert into immature granule neurons (Figure 1; Kempermann et al., 2004; Ming and Song, 2011). These precursor stages can be distinguished by their specific morphologies, expression profiles, mitotic activity and their potential to generate distinct progeny. Prototypical aNSCs (also termed radial glia-like cell or type 1) have an apical process which extends radially through the granule cell layer to the molecular layer, and express glial and stem cell markers like GFAP, nestin, BLBP, and Sox2 (Seri et al., 2001; Filippov et al., 2003; Seri et al., 2004; Suh et al., 2007; Berg et al., 2018). Besides neurons, aNSCs can generate astrocytes and daughter NSCs, suggesting multipotency and self-renewal capacity (Suh et al., 2007; Bonaguidi et al., 2011; Encinas et al., 2011; Pilz et al., 2018). In contrast to embryonic neurogenesis, aNSCs are mostly quiescent with only a few progressing through the CC at any time (Lugert et al., 2010; Encinas et al., 2011). Upon activation, aNSCs predominantly divide asymmetrically to produce another NSC and a type 2 cell (Bonaguidi et al., 2011; Encinas et al., 2011). Type 2 progenitors represent an important stage of clonal expansion and linage choice: They are transit-amplifying, prevalently using a symmetric division mode (Encinas et al., 2011; Pilz et al., 2018), and comprise cell states that mark the transition from a glial/stem-like phenotype (type 2a) to a neuronal phenotype (type 2b; Filippov et al., 2003; Steiner et al., 2006). Phenotypically, all type 2 cells are characterized by an irregularly shaped cell body with short horizontal processes, expression of nestin and Tbr2/Eomes, but no longer GFAP (Filippov et al., 2003; Hodge et al., 2008; Bonaguidi et al., 2011; Encinas et al., 2011). Whereas type 2a cells still express BLBP and Sox2, type 2b cells instead show first signs of neuronal commitment, including the expression of NeuroD1, Prox1, and DCX (Steiner et al., 2006; Kempermann et al., 2015). Committed IPCs then give rise to slowly proliferating neuroblasts that, after migrating a short distance into the granule cell layer, exit the CC to become an early postmitotic neuron (Ming and Song, 2011). New neurons then pass through a continuous process of morphological and functional maturation to fully integrate into the hippocampal network (terminally differentiated stage; Zhao et al., 2006; Toni et al., 2008; Mongiat et al., 2009; Mongiat and Schinder, 2011). However, only a small subset of the newborn cells in the SGZ will eventually develop into a mature granule neuron (Biebl et al., 2000; Tashiro et al., 2006; Sierra et al., 2010; Encinas et al., 2011). The majority of these cells are eliminated through controlled cell death, and studies suggest that two critical periods exist for their survival (Sierra et al., 2010). The main critical period is settled around the transition from IPCs to neuroblasts, when cells exit the CC and most of them die. A later critical period exists at the immature neuron stage which is only survived by neurons that are properly integrated (Tashiro et al., 2006; Sierra et al., 2010). At this time, around 4 weeks of age, adult-born neurons are highly excitable whereas inhibitory input is not yet fully established, and exhibit enhanced synaptic plasticity (Zhao et al., 2006; Mongiat et al., 2009; Marín-Burgin et al., 2012). Owing to these unique features, the young granule neurons are considered to enhance hippocampal information processing. In fact, experimental and computational evidence suggest contributions of new neurons to learning, memory, pattern discrimination, and mood regulation (reviewed in Ming and Song, 2011; Toda and Gage, 2017). Whether or not adult neurogenesis is relevant also in humans is still an ongoing debate (Kempermann et al., 2018). In consideration of two recent reports that came to opposing conclusions regarding the neurogenic potential in adult humans (Boldrini et al., 2018; Sorrells et al., 2018), it definitely deserves further efforts to elucidate the significance of this process for human DG plasticity.

### Trophic Factors and Morphogens Regulating Adult Hippocampal Neurogenesis

The adult SGZ is a specialized microenvironment that provides a wide range of extrinsic signals to preserve the self-renewing population of aNSCs and to ensure on-demand production of new granule cells. Besides the NPCs, the niche comprises a dense vascular network and several cell types, including astrocytes, endothelial cells, microglia and neurons (Massirer et al., 2011; Ming and Song, 2011; Faigle and Song, 2013; Licht and Keshet, 2015). NSCs in turn ensheath synapses and blood vessels and adhere to adjacent astrocytes with their fine processes to sense their local environment (Moss et al., 2016). As extensively reviewed by others (Suh et al., 2009; Faigle and Song, 2013; Choe et al., 2015; Toda and Gage, 2017), the effects of niche components on NSC/NPC are mediated by direct cell contacts and soluble molecules such as growth factors, morphogens, hormones, and neurotransmitters. These cues are concurrently acting on adult SGZ precursors not only to coordinate their self-renewal, expansion and fate decisions, but also to maintain NSCs in a quiescent state to prevent their premature exhaustion. Within this section we briefly recapitulate the pathways most vitally involved in CC control of NPCs and summarize their functions in adult hippocampal neurogenesis.

Brain-derived neurotrophic factor (BDNF) is known to promote adult neurogenesis through acting as an autocrine factor for dendritic maturation, functional integration and long-term survival of newborn granule cells (Bergami et al., 2008; Chan et al., 2008; Wang et al., 2015). BDNF and its receptor complex TrkB/p75NTR are also expressed by dividing SGZ progenitors (Chan et al., 2008; Li et al., 2008; Bernabeu and Longo, 2010). As shown *in vitro* and *in vivo*, increased levels of BDNF stimulate the proliferation of hippocampal NPCs, and enhance neurogenesis (Katoh-Semba et al., 2002; Li et al., 2008). It was further observed that NPC-specific deletion of TrkB impairs proliferation of DG progenitors, demonstrating that BDNF and TrkB are required to maintain basal levels of their proliferation (Li et al., 2008). Studies also suggest that increased BDNF signaling serves as mechanism through which exercise, environmental enrichment and antidepressant treatment improve hippocampal neurogenesis and cognition (Rossi et al., 2006; Li et al., 2008).

Fibroblast growth factor 2 (FGF-2) has been extensively studied *in vitro* where it is required for maintaining adult NPCs in a proliferative state (Gage et al., 1995; Gritti et al., 1996). *In vivo* studies revealed FGF-2 as potent modulator of proliferation and differentiation. For example, intraventricular administration of FGF-2 caused a strong increase in proliferation and neurogenesis in the SGZ (Jin et al., 2003; Rai et al., 2007). Moreover, the newborn neurons exhibited enhanced dendritic growth, indicating additional roles in neuronal differentiation and maturation (Rai et al., 2007; Werner et al., 2011). Increased astrocytic release of FGF-2 has recently been identified as requirement for the proliferative effects of acute stress (Kirby et al., 2013).

Insulin-like growth factor-1 (IGF-1) regulates various steps of adult SGZ neurogenesis, including proliferation, differentiation and maturation of neurons, perhaps in a dose-dependent manner (Aberg et al., 2003). IGF-1 directly stimulates proliferation and neurogenesis, both *in vitro* and *in vivo* (Aberg et al., 2000; Yuan et al., 2015). Peripheral administration of IGF-1 induces an increase of NPC proliferation through activation of their IGF-I receptors (Trejo et al., 2001; Aberg et al., 2003; Yuan et al., 2015). Moreover, the study of Trejo et al. (2001) showed that blocking brain uptake of IGF-1 completely abolishes the neurogenesis-promoting effect of voluntary exercise, suggesting that circulating IGF is an important determinant of exercise-induced changes in DG plasticity.

Vascular endothelial growth factor (VEGF) released from endothelial cells exerts direct mitogenic effects on hippocampal NPCs, as shown after intraventricular infusion of VEGF (Jin et al., 2002; Cao et al., 2004). VEGF activates quiescent aNSCs through an autocrine mechanism and VEGF signaling through VEGFR3 controls the response of aNSCs to voluntary exercise (Han et al., 2015). Congruently, blockade of VEGF signaling abolishes the neurogenic actions of running, environmental enrichment or antidepressant treatment (Cao et al., 2004; Warner-Schmidt and Duman, 2007). Altogether, previous investigations on the role of growth factors in the SGZ support a model in which they act as important mediators linking changes in environmental conditions with the processes of adult neurogenesis.

Morphogens play essential roles for neural patterning, proliferation and fate specification in the developing central nervous system. Many of these factors, like sonic hedgehog (Shh), bone morphogenetic proteins (BMPs), Wnts, and Notch continue to regulate adult NPCs. Their actions often span multiple steps of neurogenesis and differ depending on the specific cellular context. Moreover, many of these morphogen signaling cascades have been shown to cooperate with each other, adding an additional level of complexity to the control of adult neurogenesis (Shimizu et al., 2008; Antonelli et al., 2018; Armenteros et al., 2018).

Bone morphogenetic proteins released by granule neurons and NSCs are essential for maintaining the pool of undifferentiated aNSCs (Mira et al., 2010; Porlan et al., 2013). Beyond that, BMP4 signaling also decelerates the tempo of neurogenesis in later stages of the linage, by directing the transition between activation and quiescence in IPCs (Bond et al., 2014). This and other findings suggest that inhibition of BMP signaling likely represents a mechanism for rapid neuronal expansion in response to behavioral stimulation (Gobeske et al., 2009). Consistently it has been found that endogenous expression of the BMP antagonist Noggin releases NSCs from quiescence to support their proliferation, self-renewal and precursor production (Bonaguidi et al., 2008; Mira et al., 2010). Others discovered that augmented Noggin and BMP4 downregulation mediate the neurogenic and behavioral effects of antidepressants (Brooker et al., 2017). Besides that, BMPs have been shown to control glial fate decisions, having dual functions as promotor of astrogliogenesis and inhibitor of oligodendrogliogenesis (Cole et al., 2016). Accordingly, overexpression of BMP4 in the adult SGZ induces the generation of astrocytes from NSC at the expense of neurogenesis (Bonaguidi et al., 2005).

Notch signaling is reiteratively used to control cell fates during adult neurogenesis in a cell-type specific manner (Ables et al., 2011). It is well established that Notch effector genes Hes1 and Hes5 inhibit differentiation in the CNS by repressing proneural genes (Ohtsuka et al., 1999). Notch1 and Hes5 are highly expressed by aNSC, are absent from neuroblasts to become re-expressed in immature neurons (Stump et al., 2002; Breunig et al., 2007; Ehm et al., 2010; Lugert et al., 2010). Their ligands in turn are found on local astrocytes, IPCs and NSCs (Lavado et al., 2010; Lavado and Oliver, 2014). Inhibition of Notch signaling through manipulation of Notch1, Jagged1 or RBPJκ triggers the activation of quiescent NSC and the production of committed IPCs and neuroblasts, but ultimately exhausts aNSC resulting in premature depletion of their pool (Ables et al., 2010; Ehm et al., 2010; Imayoshi et al., 2010; Lugert et al., 2010; Lavado and Oliver, 2014). This demonstrates crucial roles of Notch signaling for maintaining a reserve of quiescent and undifferentiated NSC throughout life (Chapouton et al., 2010). More specifically, depletion of Jagged1-expressing committed IPCs revealed that Notch signaling serves as homeostatic feedback mechanism that links aNSC maintenance to neuronal differentiation of their progeny (Lavado et al., 2010). Later in the linage, Notch regulates the survival and dendritic morphology in the new, maturing neurons (Breunig et al., 2007).

Wnt ligands are well-established regulators of adult hippocampal neurogenesis (Varela-Nallar and Inestrosa, 2013). Secreted by local astrocytes and NPCs, Wnts act both as autocrine and as paracrine factors on the NSC niche (Lie et al., 2005; Wexler et al., 2009; Qu et al., 2010). Astrocyte-derived Wnt3 promotes neuroblast proliferation and neuronal differentiation via the canonical Wnt pathway (Lie et al., 2005). In IPCs, Wnt can directly induce the proneural gene NeuroD1 (Kuwabara et al., 2009). Accordingly, canonical Wnt signaling is required for neuronal differentiation *in vivo*, i.e., the transition of Sox2-expressing precursors into neuroblasts. In addition, Wnt signaling emerged as important pathway promoting multipotency and self-renewal of aNSCs (Mao et al., 2009). This is probably achieved through an autocrine signaling loop that has been identified *in vitro* (Wexler et al., 2009). Under conditions of low network activity, this pathway is repressed through tonic release of the inhibitor sFRP3 from granule cells, which keeps aNSCs quiescent (Jang et al., 2013). Neuronal activity decreases sFRP3 levels in the DG, resulting in aNSCs activation and accelerated maturation of new neurons, providing a mechanism to produce neurons on demand (Jang et al., 2013).

Sonic hedgehog is a member of the hedgehog family of secreted glycoproteins that acts through the patched-smoothened receptor complex to trigger the expression of GLI transcription factors (Ruiz I Altaba et al., 2002). Previous studies found that NSCs residing in the adult SGZ originate from Shh responsive cells in the ventral hippocampus and that Shh signaling is essential to establish their population (Han et al., 2008; Li et al., 2013). Although it is known that quiescent NSCs and their transit-amplifying progeny respond to Shh activity (Lai et al., 2003; Ahn and Joyner, 2005), the source of Shh in the adult DG is still obscure. Li et al. (2013) identified hilar mossy cells and neurons in the medial entorhinal cortex as principal sources of Shh in the postnatal DG. As they analyzed the mice at postnatal day 15, when the SGZ niche is almost fully established, it is conceivable that these neuronal sources sustain their activity far beyond postnatal development to provide Shh for adult SGZ precursors. Several recent reports emphasize the importance of Shh for balancing aNSC maintenance and proliferation (Lai et al., 2003; Machold et al., 2003; Bragina et al., 2010). Activation of Shh signaling elicits a strong, dose-dependent proliferative response, both *in vitro* and *in vivo* (Lai et al., 2003; Machold et al., 2003). Functional disruption of primary cilia, which are required for Shh signaling, increases CC exit in aNSCs and decreases the production of IPCs (Breunig et al., 2008; Amador-Arjona et al., 2011). A recent study showed that single copy deletion of patched1 results in deregulation at multiple steps of linage progression that accumulates with aging, including depletion of radial NSC, accumulation of IPCs, and a decrease in neurogenesis (Antonelli et al., 2018).

## Basic Principles and Essential Players of the Mammalian Cell Cycle

**FIGURE 2:**
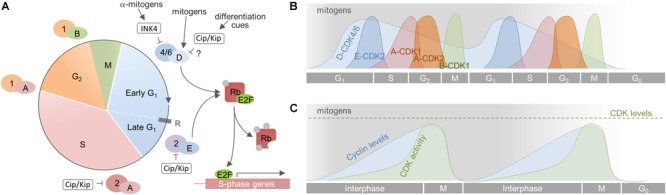
Cell cycle progression in mammalian cells. **(A)** The CC is divided into 4 phases (G_1_, S, G_2_, and M). The G_1_ phase is further divided in an early mitogen-dependent, and a late mitogen-independent phase, that are separated by the restriction point (R). Progression through the CC is driven by the sequential activation of CDKs by cyclins. The CC is initiated by mitogens [indicated by gray shading in **(B)** and **(C)**] that induce synthesis of D-cyclins, which in turn activate CDK4/6. This initiates phosphorylation of the tumor suppressor Rb that subsequently liberates E2F. This transcription factor transcribes genes necessary for the transition to S phase, including cyclin E which associates to CDK2 to fully inactivate Rb. All subsequent steps are independent from mitogen supply. **(B)** Activity dynamics of distinct cyclin-CDK complexes throughout the CC. Cyclin D-CDK4/6 complexes are active for as long as mitogens are present. Their upregulation induces expression of cyclin E and a subsequent autonomous sequence of cyclin expression and CDK activation. If mitogens are withdrawn, cyclin D-CDK4/6 complexes become inactive and the cell cannot transit through the next G_1_ Rather, and depending on the cellular context, it becomes quiescent, senescent or terminally differentiates (G_0_). **(C)** Cumulative cyclin expression and CDK activity throughout the CC. A, B, D, E – cyclins A, B, D, E; 1, 2, 4/6 – CDKs 1, 2, 4/6.

The eukaryotic CC can be divided into two major periods composed of four consecutive phases: The interphase, in which cells typically spend most of their lifetime, comprising G_1_ phase, S phase, and G_2_ phase, and the M phase in which mitosis and cytokinesis occur (Figure 2). The G_1_ phase is the longest phase and the main period of cell growth. G_1_ prepares the cell for DNA replication and cell division before it can pass into the S phase, in which the cell duplicates its chromosomes. The S phase is followed by the G_2_ phase, in which the cell proceeds growing and prepares for entering the M phase that eventually results in the generation of two daughter cells that again enter G_1_. These cells may immediately commence a new round of the CC, as is the case for rapidly dividing progenitor cells. Other cells, like slowly dividing aNSCs or terminally differentiated neurons, may exit the CC to enter a temporary (quiescent) or permanent (post-mitotic/differentiated) G_0_ state.

Unidirectional progression through the CC is driven by the coordinated activation and inactivation of cyclin-dependent kinases (CDKs) through association with cyclins and cyclin-dependent kinase inhibitors (CKIs; Figure 2). CDKs are serine/threonine kinases that require the binding of regulatory subunits, i.e., cyclins, and phosphorylation by CDK-activating kinases (CAKs) to exert their catalytic function (Sherr, 1995; Johnson and Walker, 1999; Lolli and Johnson, 2005; Malumbres, 2014). CDKs and CAKs are abundantly expressed and their levels remain fairly stable throughout the CC, consequently they are not rate-limiting for its progression (Lees, 1995; Morgan, 1995). Cyclin levels in turn fluctuate through synthesis and degradation and, in this way, periodically activate CDKs (Matsushime et al., 1994; King et al., 1996; Udvardy, 1996). The activated cyclin-CDK complexes phosphorylate multiple downstream targets, ensuring appropriate ordering of cell-cycle events (Swaffer et al., 2016). Depending on the activity of cyclin-CDK complexes in the G_1_ phase, cells will commit to divide or withdraw from CC (Blagosklonny and Pardee, 2000–2013, 2002). This decision depends on extracellular signals like mitogens and growth factors that induce the expression of D-cyclins (Matsushime et al., 1994; Sherr, 1994a; Ekholm and Reed, 2000). The D-cyclins assemble with CDK4 or CDK6 to drive progression through the G_1_ phase. Cyclin D-CDK complexes initiate the phosphorylation of Rb proteins and thus weaken their growth suppressive effect by releasing E2F transcription factors. E2Fs then start to transcribe various genes required for G_1_/S phase transition, including DNA polymerase α, cyclins E and A (Sherr, 1995; Lundberg and Weinberg, 1998; Julian and Blais, 2015). Assembly of cyclin E with CDK2 during later G_1_ further phosphorylates Rb, forming a positive feedback loop that fully activates the transcription of genes essential for DNA replication and onset of S phase (Lundberg and Weinberg, 1998; Johnson and Walker, 1999). Activation of cyclin E/CDK2 complexes demarks the point at which commitment occurs and cells do no longer require mitogens to complete division. This point, termed the restriction point, represents a point of no return and divides G_1_ into an early, mitogen-dependent phase and a late, mitogen-independent phase (Johnson and Walker, 1999; Blagosklonny and Pardee, 2000–2013, 2002). Soon thereafter, cyclin A interacts with CDK2 to turn off E2F-mediated transcription and drive the cell through S phase (Krek et al., 1994; Xu et al., 1994). In late S phase, cyclin A associates with CDK1 to facilitate transition to and progression through G_2_ and to prepare the cells entry into the M phase (Yam et al., 2002; Lindqvist et al., 2009). Finally, cyclin B builds an active complex with CDK1, termed M phase promoting factor, which coordinates the onset of M phase and the reorganization of cell structures required for mitosis and cytokinesis (Nigg, 2001; Gavet and Pines, 2010). As the newly born cells exit mitosis, cyclin B is degraded and the CC is reset, allowing the establishment of a new replication-competent state, i.e., G_1_ (King et al., 1996; Sherr and Roberts, 1999). Important to note, cyclins, CDKs and CKIs carry out important functions beyond CC regulation, either complexed or individually, such as transcription, stem cell self-renewal, differentiation, neuronal function, cell death, or metabolism (Lim and Kaldis, 2013; Pestell, 2013; Hydbring et al., 2016).

### D-Cyclins

D-cyclins are the first cyclins induced when quiescent cells become stimulated to enter the CC (Matsushime et al., 1994; Sherr, 1994a,b). Unlike other cyclins that are periodically expressed during the CC, D-cyclins are induced by extracellular mitogens, including the above mentioned niche factors (Bottazzi and Assoian, 1997; Perry et al., 1998; Ekholm and Reed, 2000; Baek et al., 2003; Frederick and Wood, 2004; Campa et al., 2008; Shimizu et al., 2008; Chen et al., 2018), and hence are regarded as crucial direct link between the extracellular environment and the CC machinery (Matsushime et al., 1994). They comprise a family of three homologous proteins (cyclin D1, D2, and D3) that bind to and thereby activate either CDK4 or CDK6 (Sherr and Roberts, 1999, 2004). Extensive studies in knockout mice uncovered that D-cyclins are, to a great extent, functionally redundant but that each has unique tissue-specific functions (Wianny et al., 1998; Sherr and Roberts, 2004; Satyanarayana and Kaldis, 2009). Intriguingly, not all cells require D-cyclins/cyclin D-CDK complexes for proliferation and embryos lacking all three D-cyclins develop normally until midgestation (Kozar et al., 2004). D-cyclins drive cells through the G_1_ restriction point, after which mitogen stimulation is no longer required to complete the cycle. They are unstable proteins that become rapidly degraded via the ubiquitin/proteasome pathway if mitogens are withdrawn (Diehl et al., 1997; Ekholm and Reed, 2000). According to the classical model of G_1_ progression, active cyclin D/CDK complexes inactivate the Rb tumor suppressor through gradual hypo-phosphorylation to liberate E2F transcription factors required for CC progression (Mittnacht et al., 1994; Sherr and Roberts, 1999). This view has been recently challenged by a study demonstrating that cyclin D-CDK complexes do not inactivate, but instead activate Rb during early G_1_ through mono-phosphorylation (Narasimha et al., 2014). Hyper-phosphorylation of Rb through Cyclin E/CDK, which inactivates Rb to ensure E2F-dependent transcription in later G_1_, seems to be independent from that mono-phosphorylation. Instead, Narashima et al. (2014) propose that Rb-mono-phosphorylation by cyclin D-CDK complexes keeps cells in an “alert” state, priming them for CC entry and preventing CC exit. The debate was fueled again by subsequent work that suggests that cyclinD-CDK4/6 complexes control the timing of onset of E2F activity, indicating that D-cyclins indeed are the main regulators of G_1_ length, rather than being the initiator of CC entry (Dong et al., 2018). Despite this controversy on being drivers or modulators of G_1_ progression, D-cyclins are consistently regarded as crucial link between extracellular growth-stimulating signals and the CC machinery. In addition to their role in modulating Rb activity, cyclin D-CDK complexes have also kinase-independent CC functions in titrating away inhibitors p21^cip1^ and p27^kip1^ from CDK2-containing complexes to facilitate cyclin E-CDK activation and coordinated CC progression in later G_1_ (Sherr and Roberts, 1999; Kozar and Sicinski, 2005).

### Cyclin-Dependent Kinases (CDKs)

To this day, eleven different CDKs have been related to the mammalian CC, out of which at least five (CDK1, 2, 3, 4, and 6) are directly involved in its progression (Satyanarayana and Kaldis, 2009; Malumbres, 2014). Contrasting many of the initial *in vitro* studies that showed specific requirements for each interphase CDK in cycle progression, mice lacking a single CDK survive, indicating profound functional redundancy among CDKs (Sherr and Roberts, 2004; Satyanarayana and Kaldis, 2009; Malumbres, 2014). Even mouse embryos lacking all interphase CDKs undergo organogenesis and develop until midgestation. This is possible because CDK1, by complexing with all necessary cyclins, can execute all crucial events required for cell division (Santamaria et al., 2007). Nevertheless, single-CDK knockout mice display more or less narrow, tissue-specific deficits demonstrating that CDKs have different roles and cannot fully compensate for each other (Sherr and Roberts, 2004; Kozar and Sicinski, 2005; Malumbres, 2014). In addition to cyclin binding, different mechanisms are engaged in the control of cyclin-CDK complexes, including phosphorylation through constitutively active CAKs and binding of CKIs (Lees, 1995; Morgan, 1995; Ekholm and Reed, 2000; Malumbres, 2014). Importantly, cyclin binding determines the timing of activation and contributes to substrate specificity of CDKs (Morgan, 1995; Satyanarayana and Kaldis, 2009).

### CDK Inhibitors (CKIs)

CDK Inhibitors are generally assumed as negative CC regulators that constrain the activities of CDKs in response to anti-mitogenic factors or starvation. Thus, CKIs in addition to D-cyclins confer a second layer of CC control by extracellular signals. They comprise two families of inhibitory proteins that differ in structure and substrate specificity (Arellano and Moreno, 1997; Sherr and Roberts, 1999; Besson et al., 2008).

Members of the INK4 (INhibitors of CDK4) family (p16^INK4a^, p15^INK4b^, p18^INK4c^, and p19^INK4d^) act as brakes of G_1_ progression in response to mitogenic withdrawal, differentiation signals and other growth inhibiting cues (Koff et al., 1993; Kato et al., 1994; Polyak et al., 1994). They bind exclusively to and inhibit CDK4 and CDK6 to prevent their interaction with cyclin D (Guan et al., 1994; Hirai et al., 1995; Sherr and Roberts, 1999; Jeffrey et al., 2000; Pei and Xiong, 2005), but with different preferences. P18^INK4c^ preferentially interacts with CDK6, whereas p16^INK4a^ associates with both CDKs (Guan et al., 1994; Noh et al., 1999). INK4 proteins differ also in their regulation and function. P15^INK4b^ is induced by growth-inhibitory factors such as TGFβ and contributes to their ability to induce growth arrest (Reynisdottir et al., 1995; Sherr and Roberts, 1999). P16^INK4a^ is ubiquitously expressed at low levels in most tissues and accumulates as cells age. It becomes induced during cellular senescence, in response to oncogenic stimuli or inactivation of Rb (Serrano, 1997; Sherr and Roberts, 1999). P18^INK4c^ and p19^INK4d^, even if sensitive to extrinsic signals, oscillate throughout the CC with maximum levels during S phase (Hirai et al., 1995; Roussel, 1999). Both are found in proliferating cells in which they facilitate CC exit during terminal differentiation (Hirai et al., 1995; Roussel, 1999). The *INK4A* locus is unique in that it transcribes another protein, p19^ARF^ that is also associated with cellular senescence (Lowe and Sherr, 2003). Instead of binding CDKs, p19^ARF^ confers its role through both, activation of p53 and inhibition of c-Myc (Quelle et al., 1995; Roussel, 1999; Qi et al., 2004).

In contrast, Cip/Kip proteins (p21^cip1^, p27^kip1^, and p57^kip2^) act more broadly by modulating the activities of cyclin D-, E- and A-dependent kinases. Cip/Kip proteins bind to preformed cyclin-CDK complexes and block their substrate access (Russo et al., 1996; Sherr and Roberts, 1999). They most effectively inhibit complexes containing CDK2 and thereby prevent S phase entry and transition (Sherr and Roberts, 1999). Discordance exists regarding their effects on cyclin D-CDK complexes. In addition to reports arguing in favor of a pan-CDK-inhibitory role of Cip/Kip proteins (Bagui et al., 2003; Pei and Xiong, 2005; Cerqueira et al., 2014), others suggest that they act as assembly and stabilization factors of cyclin D-CDK4/6 to facilitate cyclin D-dependent CC events (LaBaer et al., 1997; Cheng et al., 1999; Parry et al., 1999; Sherr and Roberts, 1999). Despite their general importance for restraining cell proliferation, each Cip/Kip protein has unique functions that distinguish it from the other family members. Expression and functional studies suggest that p21^cip1^ has a predominant role in DNA-damage-induced CC arrest, while p27^kip1^ inhibits cell growth and maintains cells in a quiescent state, and p57^kip2^ regulates growth and differentiation (Deng et al., 1995; Nakayama et al., 1996; Zhang et al., 1997; Besson et al., 2008). Unlike its siblings, p57^kip2^ expression is more restricted and under control of morphogen pathways such as Notch and BMP (Besson et al., 2008). A unique feature of p21^cip1^ and p57^kip2^ is their ability to inhibit PCNA (Zhang et al., 1993; Besson et al., 2008). This allows them to coordinately arrest the CC by both, preventing E2F-dependent transcription and inhibiting PCNA-dependent replication (Cayrol et al., 1998). During the past years Cip/Kip proteins have emerged as versatile factors with functions beyond cycle regulation, including direct transcriptional regulation (e.g., E2F and STAT3), fate determination and cell death control (Besson et al., 2008).

### Rb Proteins and E2F

Rb and its family members p107 and p130, collectively known as “pocket proteins,” are placed at the core of the molecular machinery that regulates the G_1_ restriction point (Dick and Rubin, 2013). They impose a constitutive barrier on CC progression through sequestration of E2F transcription factors, for which each Rb protein displays distinct binding preferences (Hurford et al., 1997). Rb interacts specifically with “activator” E2Fs 1–3, whereas p107 and p130 interact with “repressor” E2Fs 4 and 5 (Mulligan and Jacks, 1998; Popov and Petrov, 2014). Importantly, growing evidence suggests that E2F activities are context-dependent, e.g., switching from activators in progenitors to repressors in differentiating cells (Chong et al., 2009). Another characteristic distinguishing Rb proteins is their allocation to different CC states: while Rb is constitutively expressed in all cells, acting as *bona fide* tumor suppressor, p107 predominates in cycling cells, and p130 is most prevalent in quiescent and differentiated cells (Mulligan and Jacks, 1998; Dick and Rubin, 2013). Moreover and in contrast to Rb, p107 and p130 confer growth suppression through another mechanism involving direct inhibition of CDK2-containing complexes (Lees et al., 1992; Woo et al., 1997). Beyond their direct role in CC progression, Rb proteins can interact with many other proteins to retain cells in G_0_/G_1_, such as histone deacetylases 1 and 2, histone methyltransferases, cyclins and complexes of the SWI/SNF nucleosome remodeling complex (Brehm et al., 1998; Mulligan and Jacks, 1998; Robertson et al., 2000; Strobeck et al., 2000). In addition to their CC regulatory function, Rb and E2Fs are capable of regulating genes and processes with much broader range of function, many of which directly instruct cell fate decisions (Julian and Blais, 2015). Accordingly, Rbs and E2Fs emerged as essential regulators of stem cell fate in a number of lineages (Sage, 2012; Julian and Blais, 2015). Rb deletion is sufficient to induce CC re-entry in various cell types, suggesting that it helps to maintain quiescence also in aNSCs (Sage et al., 2003; Sage, 2012). Furthermore Rb’s capacity to modulate chromatin structure has been proposed as important regulator of stem cell plasticity (Chinnam and Goodrich, 2011; Sage, 2012).

## Specific Roles of Cell Cycle Components in Regulating Adult Hippocampal Neurogenesis

### D-Cyclins

All three D-cyclins are dynamically expressed during neurulation (Wianny et al., 1998). Later in brain development, D3 becomes gradually lost, whereas D1 and D2 activities are maintained to regulate the generation of distinct progenitor populations (Sun et al., 1996; Bryja et al., 2004; Glickstein et al., 2007a; Komada et al., 2013). Recent studies suggest that, beside their role in proliferation, D-cyclins and D1 in particular may directly control stem cell fate decisions to induce neuronal differentiation (Lukaszewicz and Anderson, 2011; Pauklin and Vallier, 2013; Pauklin et al., 2016). Apparently, this is accomplished by two complementary mechanisms: D-cyclins cross-talk with the Activin/Nodal–Smad2/3 signaling pathway through CDK4/6 and also directly bind to transcription factors that suppress endoderm and activate neuroectoderm genes in stem cells (Pauklin and Vallier, 2013; Pauklin et al., 2016). Further, D1 has been identified as critical component of FGF-2 and Wnt signaling that inhibits astroglial differentiation of NSCs (Bizen et al., 2014).

During *embryogenesis*, D1 and D2 appear to have complementary roles in proliferation and differentiation of NPCs (Glickstein et al., 2007a,b, 2009; Lukaszewicz and Anderson, 2011). Initially it was shown that D1 and D2 define separate progenitor pools in the embryonic neocortex (Ross, 1996; Glickstein et al., 2007a). Whereas D1 is predominantly expressed in the VZ, D2 is localized mainly to the SVZ (Glickstein et al., 2007a). More detailed studies revealed that D1 and D2 co-label subsets of Pax6 expressing radial glia/apical precursors, which are the stem cells in the embryonic brain (Glickstein et al., 2009). As these cells commit to the neuronal linage, D1 is gradually downregulated and D2 becomes induced (Glickstein et al., 2009; Lange et al., 2009). The selective expression of D2 in Tbr2-positive IPCs/basal precursors and their loss upon genetic deletion of D2 suggest that D2 is selectively used for the expansion of the IPC pool (Glickstein et al., 2009). Withal, the knockout also diminishes the radial glia population. The same study revealed severe disturbances in CC dynamics in progenitors of D2 mutants, such as lengthening of G_1_, decreased in-cycle and increased cycle-exit fraction of IPCs, consistent with premature terminal differentiation. Interestingly, deletion of D2 not only altered G_1_ length but it also shortened the S phase. In contrast, CC parameters and numbers of Pax6 and Tbr2-positive precursors in the cortical neuroepithelium remained unchanged in D1 mutants (Chen et al., 2005; Glickstein et al., 2007a, 2009). This shows that D2, perhaps in conjunction with other CC proteins, can compensate for the loss of D1 but not vice versa (Carthon et al., 2005; Chen et al., 2005). Collectively, these findings show that D2 is more essential for cortical development than D1. They fit a concept in which D2 is required for expansive divisions of radial glia and IPCs whereas D1 drives asymmetric stem cell divisions to maintain their pool (Glickstein et al., 2009). Supporting the progenitor-specific requirement of D2 in brain formation, D2 mutant mice also display a disproportionate loss of parvalbuminergic interneurons in the neocortex and hippocampus, as well as loss of half of cerebellar granule neurons and virtually all cerebellar stellate interneurons (Huard et al., 1999; Glickstein et al., 2007b; Leto et al., 2011). Direct *in vivo* evidence for a CC-independent proneurogenic function of cyclin D1, as originally discovered in human pluripotent stem cells (Pauklin and Vallier, 2013; Pauklin et al., 2016), comes from loss- and gain-of-function experiments in the embryonic spinal cord, where D1 is expressed by differentiating NPCs and newly generated neurons (Lukaszewicz and Anderson, 2011). Knockdown of D1, but not that of D2, decreased neuronal fate specification and differentiation through activating the non-canonical Notch effector Hes6, whereas overexpression had the opposite effect. D2, on the other hand, promotes proliferation and maintains the undifferentiated progenitor pool, probably through regulation of the canonical Notch effector Hes5 (Lukaszewicz and Anderson, 2011). Strikingly, studies in the embryonic cortex have shown that asymmetrically inherited D2 may act as fate determinant in radial glia of the VZ (Tsunekawa et al., 2012). During division, these cells allocate D2 mRNA to their basal end-feet (Glickstein et al., 2007a; Tsunekawa et al., 2012). The daughter inheriting the basal process and thus D2 upon asymmetric mitosis will become another radial glia cell that maintains self-renewal capacity, while its sibling will differentiate into an IPC or a neuron (Tsunekawa et al., 2012). The molecular mechanisms are not fully understood, but the biased localization of D2 to one daughter might shorten her G_1_ phase. Alternatively, D2 might control cell fate independent from its direct action on CC progression. D2 might advance proliferation and inhibit neurogenesis through elimination of the CKI p27^kip1^, which promotes neuronal differentiation (Nguyen et al., 2006; Susaki et al., 2007; Tsunekawa et al., 2012). In opposition to neocortical development, both D-cyclins are expressed in the VZ of the early hippocampal primordium (Glickstein et al., 2007a). At perinatal ages, they are also found in the dentate anlage, but D1 predominates in the secondary and tertiary matrices, while more D2-positive neuroblasts are found in the dentate migratory stream. Postnatally, D1 and D2 are found in the tertiary matrix and the emerging SGZ. In the adult DG, D2 expression is restricted to the SGZ whereas D1 shows a more widespread, scattered expression throughout all layers of the DG (Kowalczyk et al., 2004; Glickstein et al., 2007a).

In accordance with the apparently distinct roles of cyclins D1 and D2 during development, absence of either cyclin is associated with strikingly different outcomes in the *adult brain*. As mentioned before, mice lacking D1 have essentially normal brain sizes with no selective deficits in particular regions, except a cerebellar hypoplasia (Chen et al., 2005; Pogoriler et al., 2006; Glickstein et al., 2007b, 2009). The gross architecture of D2 mutant brains is also normal, but these mice are microcephalic (about 25% reduced size), and especially the cortex, cerebellum, olfactory bulb and hippocampus are smaller in size (Kowalczyk et al., 2004; Glickstein et al., 2009; Ansorg et al., 2012). The lack of apparent deficits in D1 knockout mice may result from compensatory mechanisms, including the upregulation of other G_1_ cyclins, i.e., cyclin D2 and cyclin E (Carthon et al., 2005; Chen et al., 2005; Glickstein et al., 2007a).

**Cyclin D1** is expressed in the adult SGZ. Some of the D1 cells proliferate as indicated by incorporation of the S phase label bromodeoxyuridine (Glickstein et al., 2007a). Studies evaluating the consequences of D1 deletion on hippocampal cell proliferation came to conflicting results, with one showing no effect on proliferation, and another reporting >40% reduction of BrdU incorporation into the adult SGZ (Kowalczyk et al., 2004; Ma et al., 2010). In support of a role of D1 in aNPC proliferation, Ma et al. (2010) found that cultured precursor cells of D1 knockout mice become arrested in G_0_/G_1_. In addition, mutated precursors displayed increased apoptosis and disturbed differentiation into astrocytes, while neuronal differentiation remained intact (Ma et al., 2010). Yet, the overall picture of D1 regulation of adult neurogenesis is far from clear and needs further investigation. For instance, none of the previous studies had looked on the specific requirements of D1 in distinct precursor types as has been published for the embryonic brain. Evaluating such roles is complicated through the fact that D2 becomes upregulated and might compensate in the SGZ of D1 mutant mice (Carthon et al., 2005; Glickstein et al., 2007a).

Available data on the role of **cyclin D2** in adult hippocampal neurogenesis are more consistent. The SGZ of adult D2-deficient mice is virtually devoid of proliferating cells and neuroblasts (Kowalczyk et al., 2004; Ansorg et al., 2012). We showed that the requirement of D2 for adult neurogenesis builds up progressively during postnatal development. With full maturity of the DG/SGZ around 4 weeks of age, mutant mice are virtually devoid of newly born neurons in this region (Altman and Bayer, 1990; Ansorg et al., 2012; Nicola et al., 2015). Moreover, this impairment in adult neurogenesis cannot be overcome through exposure to physiological or pharmacological stimuli that normally increase adult hippocampal neurogenesis. For instance, numbers of cells incorporating BrdU remained negligible in response to environmental enrichment or fluoxetine treatment (Kowalczyk et al., 2004; Jedynak et al., 2012). So far it is not clear whether this deficit results from the inability of mutant SGZ precursors to divide or from a lack of precursors that can respond to such neurogenic stimuli (Ansorg et al., 2012). Interestingly, despite of their severe impairments in adult neurogenesis, D2KO mice are able to master hippocampus-dependent tasks, including contextual or trace fear conditioning, object recognition, and spatial learning in Barnes or water mazes (Jaholkowski et al., 2009; Jedynak et al., 2012; Ben Abdallah et al., 2013; Urbach et al., 2013). However, impairments became apparent in tasks requiring a certain degree of flexibility for integrating novel information into previously learned contexts (Garthe et al., 2014). Clinically relevant, loss-of-function mutations affecting the *ccnD2* locus have been linked to brain pathologies like microcephaly and epilepsy (by loss of interneurons; Glickstein et al., 2009). In humans, *de novo ccnD2* mutations stabilizing D2 have been identified as cause of the megalencephaly polymicrogyria-polydactyly hydrocephalus syndrome (Mirzaa et al., 2014).

In summary, available data suggest that D1 and D2 have discrete, non-overlapping functions in adult neurogenesis. Considering recent observations in human embryonic or pluripotent stem cells, available data suggest that the main function of D2 is controlling adult precursor proliferation, whereas D1 seems to be rather involved in neuronal differentiation (Lukaszewicz and Anderson, 2011; Pauklin and Vallier, 2013; Pauklin et al., 2016).

### Cyclin-Dependent Kinases (CDKs)

All three G_1_ CDKs have been detected in apical and basal precursors of the embryonic VZ/SVZ (Telley et al., 2016). However, only combinatorial ablation of two G_1_ CDKs affected cortical progenitor proliferation *in vivo*, suggesting redundant or compensatory functions of CDKs during corticogenesis (Lim and Kaldis, 2013; Grison et al., 2018). Moreover, the effects of CDK2/4, CDK2/6, or CDK4/6 double-knockouts were progenitor type-dependent, indicating that discrete combinations of CDKs are used in distinct precursor populations (Lim and Kaldis, 2013; Grison et al., 2018).

**CDK2** kinase activity is high during *cerebral development* but drops rapidly thereafter (Carey et al., 2002). This suggests important functions of this enzyme in cortex development, which has been corroborated by several *in vitro* studies. For instance, cultured cortical precursors increased CDK2 activity upon FGF-2 stimulation (Lukaszewicz et al., 2002; Li and Dicicco-Bloom, 2004). Also, inhibition of CDK2 activity through adenoviral delivery of double-negative CDK2 mutants induced complete growth arrest in isolated NSC from embryonic forebrain (Ferguson et al., 2000). Nevertheless, no developmental deficits or impaired proliferation were observed in the SVZ of CDK2 mutant mice (Jablonska et al., 2007; Grison et al., 2018). Even the concomitant loss of CDK6 had no effect on the proliferation rate of cortical precursors, suggesting functional compensation by CDK1, CDK4 or other CDKs. Indeed, a subsequent study found altered CC parameters and increased neurogenic divisions in NSCs of CDK2/CDK4 double knockout embryos (Lim and Kaldis, 2012). This prevented the expansion of the basal IPC pool culminating in a striking thinning of the embryonic cortex. Nevertheless, isolated embryonic precursors of these mice retained their capacity to divide due to compensatory upregulation of CDK1 (Lim and Kaldis, 2012). CDK2 appears also dispensable in the neurogenic niches of the *adult brain*. Functional deletion of CDKs had neither an effect on proliferation, nor on differentiation or survival of hippocampal NPCs, both under basal and under seizure conditions (Vandenbosch et al., 2007). Others showed that CDK2 is expendable for proliferation of NSCs and neuroblasts in the adult SVZ (Jablonska et al., 2007; Caillava et al., 2011). Instead, these loss-of-function studies revealed an involvement of CDK2 in the proliferation, lineage commitment and differentiation of oligodendrocyte precursors in the adult SVZ (Jablonska et al., 2007; Caillava et al., 2011).

Two recent studies indicate that **CDK4** drives IPC expansion in the *embryonic* SVZ between E13.3 and E15.5 (Lim and Kaldis, 2013; Grison et al., 2018). This, however, became only apparent if CDK4 was depleted together with another G_1_ CDK, i.e., CDK2 or CDK6. Interestingly, any of these combinatorial depletions left the population of apical NSCs in the VZ intact (Lim and Kaldis, 2013; Grison et al., 2018). Yet, adult single mutants are microcephalic (Beukelaers et al., 2011), pointing toward important non-redundant functions of CDK4 at developmental time points not investigated in the before mentioned studies. CDK4 is also expressed in dividing precursors of the *adult neurogenic niches* (Beukelaers et al., 2011), but like during development the picture is far from clear. Initial functional evidence came from loss- and gain-of-function experiments that described an involvement of cyclin D-CDK4 in fate specification of embryonic and adult NSCs (Lange et al., 2009; Artegiani et al., 2011). They observed that overexpression of cyclin D1-CDK4 reduces G_1_ length and neurogenesis in the adult SGZ as well as in the embryonic cortex, while RNAi-mediated silencing of cyclin D1-CDK4 lengthens G_1_ and increases neuronal differentiation during corticogenesis. However, Calegari and his group manipulated CDK4 together with D1, so no clear conclusions on the role of either of them can be drawn from these studies (which, by the way, was not the their aim). Rather, they provided persuasive evidence for a link between G_1_ length and differentiation of NSC (Lange and Calegari, 2010; Salomoni and Calegari, 2010). More direct evidence was presented by subsequent *in vitro* studies (Roccio et al., 2013; Chirivella et al., 2017). Inhibition of CDK4 in precursors isolated from the adult SGZ or SVZ proportionally increased the number of cells in G_1_ and forced neuronal differentiation. Recently, activation of CDK4 has been identified as mechanism of the proliferative effect of insulin in aNSC (Chirivella et al., 2017). Unexpectedly, this study also implies that CDK4 activity in response to insulin can promote terminal differentiation of NSCs. Yet, CDK4 appears to be dispensable *in vivo*. Although CDK4 is expressed by virtually all Ki67-positive cells in the adult SGZ, its loss had no effect on CC parameters, proliferation or neurogenesis (Beukelaers et al., 2011).

Available evidence for a role of **CDK6** in *brain development* is more or less indirect. A study in mice revealed that downregulation of CDK6 by Gli3 is required for the scheduled onset of cortical neurogenesis (Hasenpusch-Theil et al., 2018). In humans, mutations in the *cdk6* locus have been linked to autosomal recessive primary microcephaly (Naveed et al., 2018), suggesting important functions in the expansion of cortical precursor cells. However, brains of CDK6 knockout mice exhibit no obvious phenotype despite smaller olfactory bulbs (Beukelaers et al., 2011), and even the concomitant deletion of CDK2 did not interfere with proliferation of cortical progenitors in mouse embryos (Grison et al., 2018).

In the *adult SGZ*, CDK6 displays essentially the same expression pattern as CDK4 (Beukelaers et al., 2011). Genetic inactivation of CDK6 reduced proliferation of SGZ precursors by >50%. Loss of CDK6 specifically prevents the expansion of neuronally committed precursors by lengthening G_1_ phase duration and premature CC exit, resulting in decreased neurogenesis (Beukelaers et al., 2011). The group of Caron et al. (2018) then asked for the mechanism of CDK action in adult SGZ precursors. By generating mice bearing a kinase-dead allele of CDK6 they demonstrated that the function of CDK6 in hippocampal neurogenesis relies essentially on its kinase activity. They also observed that p27^cip1^ is the main inhibitor of CDK6 activity in hippocampal progenitors.

Altogether, even if both early G_1_ phase CDKs are widely expressed in the adult neurogenic niches, available data imply that they are not functionally redundant. Whereas CDK4 appears to be the prevailing CDK during corticogenesis, CDK6 exerts important functions in the adult DG. It needs to be determined whether the distinct outcomes of manipulating CDK4 and CDK6 are caused by intrinsically different functions of CDK4 and CDK6 (e.g., as transcription factor or through site-specific mono-phosphorylation of Rb), by selective interactions with regulatory D-cyclins, CAK and CKIs, or other mechanisms (Grossel and Hinds, 2006; Bryja et al., 2008; Bockstaele et al., 2009; Kollmann et al., 2013). In support of the first, it has been shown that CDK4 and CDK6 phosphorylate Rb with different residue selectivity (Takaki et al., 2005; Satyanarayana and Kaldis, 2009). In addition to its role in proliferation, findings suggest that CDK6 prevents terminal differentiation in a variety of cell types. This function, which involves phosphorylation of fate-determining transcription factors, is not shared with CDK4 (Grossel and Hinds, 2006). For instance, expression of CDK6, but not CDK4, in primary astrocytes resulted in expression of progenitor cell markers and dedifferentiation (Ericson et al., 2003). Moreover, CDKs can directly inhibit pro-neuronal transcription factors as demonstrated in frog eggs (Ali et al., 2011). There, rising CDK levels quantitatively phosphorylate Ngn2 on multiple sites which inhibits neuronal differentiation.

### CDK Inhibitors (CKIs)

**INK4 family members** are dynamically expressed during mouse development and adulthood (Zindy et al., 1997a,b; Canepa et al., 2007). P15^INK4b^ and p16^INK4a^ are first detected postnatally. Whereas p16^INK4a^ becomes upregulated in all organs as mice age, p15^INK4b^ is more restricted and lacking from brain (Zindy et al., 1997a,b). It is assumed that the ubiquitous increase in p16^INK4a^ is important for preventing neoplastic transformation in later life (Serrano et al., 1996). On the contrary, p18^INK4c^ and p19^INK4d^ are steadily expressed during development and adulthood (Zindy et al., 1997a). Both are detectable also in embryonic and postnatal brains, albeit in disparate patterns (Zindy et al., 1997b).

In the developing cortex, **p18^***INK4c***^** is restricted to proliferating neuroblasts around the time when they lengthen G_1_ (Zindy et al., 1997b). Postnatally, p18^INK4c^ is expressed by neuroblasts of the DG, but falls below detectable levels with reaching adulthood (Zindy et al., 1997b). Accordingly, loss of p18^INK4c^ did neither impair proliferation nor neurogenesis in the adult SGZ (Caron et al., 2018).

**P19^***INK4d***^**, on the other hand, is highly expressed in adult brain (Zindy et al., 1997a,b). However, p19^INK4d^ is restricted to postmitotic neurons, independent of developmental age (Zindy et al., 1997a,b). Here, it actively represses CC re-entry together with the Cip/Kip family member p27^kip1^ (Zindy et al., 1997b).

**P16^***INK4a***^** is the only INK4 family member displaying an essential role in adult neurogenesis. Supporting the expression data from embryonic brain, deletion of p16^INK4a^ had no obvious effect on brain development nor on proliferation and self-renewal capacity of NSCs from young adult mice, both, *in vivo* and *in vitro* (Bachoo et al., 2002; Molofsky et al., 2006). In the *aging SVZ*, however, studies show that accumulation of p16^INK4a^ contributes to the progressive decline of NSC function (Molofsky et al., 2006; Nishino et al., 2008). Deletion of p16^INK4a^ partially reverses this phenotype by increasing both, the self-renewal capacity of NSCs and precursor proliferation in the SVZ of aged mice (Molofsky et al., 2006). In support of these findings, overexpression of p16^INK4a^ in NSCs from the embryonic SVZ strongly impairs their self-renewal capacity (Nagao et al., 2008). On the contrary, studies found no evidence for an involvement of p16^INK4a^ in the aging-related decline in *adult hippocampal neurogenesis* (Molofsky et al., 2006). Rather, as demonstrated in a very recent report, p16^INK4a^ appears to prevent aNSC’s release from quiescence when neurogenic stimuli are present (Micheli et al., 2019). They showed that in the DG of middle-aged p16^INK4a^ knockout mice, running highly increased aNSC numbers as well as IPCs through forcing their entry to CC, suggesting that p16^INK4a^ plays a role in the maintenance of aNSCs after a neurogenic stimulus, to keep a reserve of their self-renewal capacity during aging (Micheli et al., 2019). Moreover, p16^INK4a^ deletion counteracts the disruption of DG precursor proliferation after irradiation, demonstrating that p16^INK4a^ expression is a mechanism mediating the radiation-induced loss of neurogenesis (Le et al., 2018). Another study showed that p16^INK4a^ acts as a barrier to direct neuronal transdifferentiation and functions in the linage-restriction of astrocytes (Price et al., 2014). Altogether, in the light of INK4 family members being the main inhibitors of CDK4/6, their physiological significance in adult neurogenesis appears comparably low, with the exception of p16^INK4a^ that emerged as important regulator of NSC self-renewal in the aging brain. However, whereas the aging-related rise in p16^INK4a^ expression contributes to the progressive decline of NSC function and regenerative capacity in the SVZ, it helps to maintain the pool of quiescent NSCs in the DG through protecting them from excessive activation by neurogenic stimuli.

In accordance with their wider range of CDK inhibitory activity as compared to INK4 proteins, the **Cip/Kip family members** are vitally involved in a broad spectrum of cell fates, which is even extended through a range of CC-independent actions. The three Cip/Kip CKIs have distinct and overlapping functions, that either are surprisingly stable between different niches (e.g., for p27) or vary depending on time and context (e.g., for p21).

Contrary to p27^kip1^ and p57^kip2^, **p21^***cip1***^** expression in the developing cortex is weak, suggesting it plays no major role during development (Van Lookeren Campagne and Gill, 1998; Tury et al., 2011). P21^cip1^ is expressed in the stem cell niches of the adult brain but, as seen for many other CC regulators, it has regionally distinct functions. In the *adult SVZ*, p21^cip1^ is required to balance quiescence, proliferation and differentiation of NSCs (Kippin et al., 2005; Marques-Torrejon et al., 2013; Porlan et al., 2013). Loss of p21^cip1^ results in CC shortening and cumulative hyperproliferation of aNSCs, leading to impaired long-term self-renewal and premature exhaustion of NSCs in aged mice (Kippin et al., 2005). The knockout had no effect on the proliferation of IPC, suggesting that the functions of p21^cip1^ are highly specific for aNSCs. The mechanisms involved in p21^cip1^-dependent regulation of NSCs were evaluated by two subsequent studies (Marques-Torrejon et al., 2013; Porlan et al., 2013). The first found that p21^cip1^ acts as transcriptional repressor of *Sox2* in aNSCs, thereby preventing replicative stress and exhaustion of the NSC pool (Marques-Torrejon et al., 2013). Later, the same group reported that p21^cip1^ maintains aNSC in an undifferentiated, multipotent state through transcriptional repression *Bmp2* (Porlan et al., 2013). Strikingly, p21^cip1^-dependent transcriptional regulation of both genes emerged to be independent from its role as CDK inhibitor and CC regulator. Rather, p21^cip1^ represses *Sox2* through direct interaction with the *Sox2* enhancer, whereas it modulates *Bmp2* expression through association with E2F (Porlan et al., 2013). These data demonstrate that p21^cip1^ acts in distinct ways to link the relative quiescence of aNSCs to their longevity and potentiality (Porlan et al., 2013). P21^cip1^ is also expressed at high levels in the *adult SGZ*. There, it is expressed by IPCs, neuroblasts and immature neurons, but absent from radial glia-like NSC and mature granule cells (Pechnick et al., 2008, 2011). Accordingly, several studies show that committed IPCs or neuroblasts divide more actively in the DG of p21^cip1^ knockout mice, suggesting that p21^cip1^ restrains their proliferation (Pechnick et al., 2008, 2011; Zonis et al., 2013; Li and Wong, 2018). Another study, despite not reproducing the effects of p21^cip1^ deletion in native DG, identified p21^cip1^ as intrinsic suppressor of precursor proliferation after ischemic brain injury (Qiu et al., 2004). Given its importance for restricting adult neurogenesis, it is not surprising that p21^cip1^ is relevant also in neuropathological contexts. For instance, the studies of Pechnick et al. (2008, 2011) revealed that antidepressants exert their beneficial effects on hippocampal neurogenesis and behavior through down-regulation of p21^cip1^. Another report suggests that an increase of p21^cip1^ is responsible for the CC arrest of neuroblasts in response to acute systemic inflammation (Zonis et al., 2013). Together these studies again highlight the differences in the neurogenic niches of the adult brain, showing that p21^cip1^ serves to maintain a population of undifferentiated NSCs in the SVZ, whereas it acts as brake for proliferation of later stages of neuron development in the SGZ.

Several studies have demonstrated the importance of the Cip/Kip family members **p27**^***kip1***^ **and** **p57**^***kip2***^ as regulators of CC exit, differentiation and migration (Tury et al., 2012). Expression profiles and observations in mutant mice suggest that they regulate different sets of precursor cells in the *embryonic cortex* (Tury et al., 2012). Besides being co-expressed with p57^kip2^ in the cortical plate, **P27^***kip1***^** is exclusively found in IPCs of the SVZ (Van Lookeren Campagne and Gill, 1998; Mairet-Coello et al., 2012). In this way, genetic manipulation assays revealed that p27^kip1^ promotes CC exit exclusively in IPCs (Goto et al., 2004; Tarui et al., 2005; Nguyen et al., 2006; Mairet-Coello et al., 2012; Clement et al., 2017). Moreover, they identified roles of p27^kip1^ in precursor differentiation and migration of cortical neurons (Nguyen et al., 2006; Tury et al., 2011; Hasan et al., 2013; Clement et al., 2017). These effects are independent from its CC activity, as neuronal differentiation is driven through stabilization of Ngn2 and migration is controlled through inhibition of RhoA signaling (Vernon et al., 2003; Nguyen et al., 2006).

In the *adult SVZ*, p27^kip1^ has very similar functions as during cortex development. As exemplified in p27^kip1^-deficient mice, p27^kip1^ specifically promotes CC exit of IPCs, while playing no role in NSCs (Doetsch et al., 2002; Gil-Perotin et al., 2011). Cells originating in the adult SVZ then migrate a long distance via the rostral migratory stream toward the olfactory bulb to replace resident granule neurons (Doetsch et al., 1997). P27^kip1^ is ubiquitously expressed in these areas to prevent the proliferation of newborn neurons (Li et al., 2009). The size of the rostral migratory stream is increased and olfactory bulb development is delayed in p27^kip1^ knockout mice, which may reflect a migration deficit (Li et al., 2009).

Two types of p27^kip1^-positive cells exist in the *adult DG*, strongly positive cells in the SGZ and weakly positive postmitotic neurons in the GCL and hilus (Qiu et al., 2009; Beukelaers et al., 2011; Andreu et al., 2015). Detailed immunophenotyping revealed that p27^kip1^ is expressed by virtually all type 1 NSCs, in the majority of IPCs, in neuroblasts and immature neurons (Qiu et al., 2009; Andreu et al., 2015). Deletion of p27^kip1^ increases proliferation of radial aNSC *in vivo* and *in vitro*, and Ki67 can be detected only in those few aNSCs that are p27^kip1^-negative, demonstrating a role of p27^kip1^ in aNSC quiescence (Qiu et al., 2009; Andreu et al., 2015; Horster et al., 2017). Activation of p27^kip1^ mutant NSCs has no effect on the total size of the aNSC pool, indicating that p27^kip1^ has no role in the choice between symmetric and asymmetric aNSC divisions (Andreu et al., 2015). Mechanistically, p27^kip1^ acts downstream of BMP4 to maintain aNSC quiescent (Andreu et al., 2015). Conversely, p27^kip1^ is low in transit-amplifying type 2a cells that accordingly are resistant to deletion of p27^kip1^ (Andreu et al., 2015). Later in the linage, p27^kip1^ deletion delays the CC exit of neuroblasts, resulting in a net increase in newborn neurons, just as in the aSVZ (Qiu et al., 2009; Andreu et al., 2015). Summing up, these studies demonstrate that p27^kip1^ acts as dual modulator of both aNSC quiescence and terminal CC exit of immature neurons. Recent studies also provided some insight into the role of p27^kip1^ in differentiation. P27^kip1^ increases upon neuronal differentiation *in vitro* and *in vivo* (Varodayan et al., 2009; Andreu et al., 2015; Horster et al., 2017). It becomes induced by proneural genes, such as NeuroD2 or Mash1, suggesting that p27^kip1^ is employed by neural determination factors to force differentiation and CC exit (Farah et al., 2000). Others showed that selective phosphorylation and stabilization of p27^Kip1^ by CDK5 is crucial to neuronal differentiation of NSCs and promotes neurite outgrowth as neurons differentiate (Zheng et al., 2010). The role of p27^Kip1^ in neuronal differentiation is further strengthened by observations in embryonic and pluripotent stem cells, in which 27^Kip1^ promotes neuronal differentiation through both stabilization of Ngn2 and repression of *Sox2* (Nguyen et al., 2006; Li et al., 2012).

**P57^***kip2***^** is expressed in cycling precursors of the *embryonic* VZ and SVZ (Ye et al., 2009; Mairet-Coello et al., 2012). Studies in mutant mice revealed that p57^kip2^ controls CC length and proliferation both in radial NSCs and IPCs (Tury et al., 2011; Mairet-Coello et al., 2012). Accordingly, it has been shown that p57^kip2^ knockout mice display macrocephaly due to embryonically increased proliferation of radial glia NSCs (Mairet-Coello et al., 2012). In promoting neuronal differentiation p57^kip2^ is even more effective than p27^kip1^ (Tury et al., 2011). And similar to p27^kip1^, p57^kip2^ regulates migration in the developing cortex, albeit at later stages (Nguyen et al., 2006; Tury et al., 2011). A feature that clearly distinguishes the two CKIs is the suppressive action of p57^kip2^ on astrogliogenic fate decisions (Tury et al., 2011). Noteworthy, all effects of p57^kip2^ on neurogenesis mentioned so far require their interaction with cyclin/CDK complexes.

Information on the role of p57^kip2^ in the *adult DG* is unfortunately sparse. Furutachi et al. (2013) show that conditional deletion of p57^kip2^ in aNSCs activates their proliferation and increases neurogenesis both in young and aged mice. Prolonged deletion, however, led to depletion of aNSCs and reduced neurogenesis (Furutachi et al., 2013). They further observed that the running-induced increase in aNSC proliferation, which is accompanied by a decreased expression of p57^kip2^ in wildtypes, is impaired in p57^kip2^ mutants. Their results suggest that p57^kip2^ maintains radial aNSCs in a quiescent state to preserve a pool of recruitable NSCs throughout life and that the reduction of p57^kip2^ is required for activation of aNSCs by a neurogenic stimulus such as running. In addition, suppression of p57^kip2^ directs NSCs isolated from the aSGZ toward the oligrodendrocyte linage while reducing astroglial characteristics, suggesting an involvement in linage commitment or maintenance of stem cell character (Jadasz et al., 2012).

### Rb Proteins and E2F

In agreement with research reporting Rb functions in diverse cellular pathways of various cell types, recent studies identified distinct requirements for **Rb/E2F** in the brain, including cell division, differentiation and migration of precursor cells. During brain development, telencephalon-specific or heterozygous Rb mutants displayed increased and ectopic division of neuroblasts, demonstrating a requirement of Rb for CC exit after commitment to a neuronal fate (Lee et al., 1994; Callaghan et al., 1999; Ferguson et al., 2002). Ectopic division may be supported through the concomitant increase in FGF-2 levels in the VZ and cortical plate of the mutant mice (McClellan et al., 2009). Others identified severe deficits in the proliferation, maturation and subsequent tangential migration of specific interneuron subtypes in these mice, suggesting essential involvement of Rb in differentiation and migration of cortical precursor cells (Ferguson et al., 2005; Ghanem et al., 2012). Rb overexpression in cultured NSCs from newborn rats increased their differentiation – according to the specification factor added – into neurons, astroglia or oligodendrocytes (Jori et al., 2007). This suggests a requirement of Rb in determination of NSC fate in response to extracellular linage specification signals. A recent study examined DG development and *adult hippocampal neurogenesis* in conditional Rb knockout mice, essentially describing similar defects as observed in the embryonic cortex (Vandenbosch et al., 2016). Both, in the developing and adult DG they observed delayed CC exit and increased ectopic proliferation of neuronally committed progenitors and neuroblasts. However, the increased neuron birth was counteracted by an increase in cell death, which resulted in a long-term reduction of neurogenesis in the adult DG. Consistent with these findings, Rb-deletion in the aSVZ and rostral migratory stream was associated with increased progenitor proliferation and neurogenesis but impaired long-term survival (Naser et al., 2016). Together, these studies suggest conserved requirements for Rb in embryonic and adult neurogenic niches, i.e., exit from CC, restriction of proliferation and survival of newborn neurons (Vandenbosch et al., 2016). Consistent with prior findings in hematopoietic stem cells, none of the studies noticed consequences of the Rb knockout in the NSC population, suggesting that other Rb proteins are responsible for the control of self-renewal and quiescence in NSCs (Vanderluit et al., 2004; Walkley and Orkin, 2006; Naser et al., 2016; Vandenbosch et al., 2016).

Current knowledge on the role of **p107** in neurogenesis relies only on studies conducted in the embryonic VZ and adult SVZ, in which it is highly expressed (Vanderluit et al., 2004, 2007). Contrary to Rb, p107 is expressed in uncommitted precursors and becomes downregulated as they commence to differentiate (Callaghan et al., 1999; Vanderluit et al., 2004). P107 knockout mice display increased proliferation of progenitors and slowly dividing NSCs *in vivo* that exhibit an increased self-renewal potential in *in vitro* (Vanderluit et al., 2004, 2007). Concomitantly, effectors of the Notch pathway including Notch1, Dll1, and Hes1 become upregulated in the mutated cells, suggesting that p107 controls the NPC population through suppression of Notch activity (Vanderluit et al., 2004, 2007). Indeed, p107 was shown to directly interact with regulatory sequences of Notch1 and Hes1, and accordingly the phenotype of p107 mutants could be rescued through deletion of Hes1 (Vanderluit et al., 2007). Nevertheless, p107 knockout mice ultimately display reduced neurogenesis due to decreased differentiation and increased apoptosis of uncommitted progenitor cells (Vanderluit et al., 2007). As reported by others, the p107/E2F pathway controls NPC division also through restricting the autocrine production of growth factors such as FGF-2 (McClellan et al., 2009). Together, these studies identified distinct mechanisms by which p107 regulates the expansion and neuronal commitment of stem and progenitor cells in the embryonic and adult brain.

Also, little is known about the role of **p130**, which is predominantly expressed in quiescent and terminally differentiated cells, during adult neurogenesis (Mulligan and Jacks, 1998). View lines of evidence suggest an involvement in NSC differentiation. For instance, p130 forms complexes with E2F4 and SIN3A to repress *Sox2* transcription during iPSC differentiation (Li et al., 2012). Additionally, p130 overexpression in differentiating NSCs isolated from newborn rats causes a shift toward the cell type induced by differentiation cues, similar to the effects of Rb overexpression. No such effect was observed under proliferating conditions, suggesting that p130 and Rb reinforce the cellular responses of NSCs to extracellular fate specification signals (Jori et al., 2007).

Together, these studies exemplified diverse functions of distinct Rb proteins in the regulation of neural stem and progenitor cells, not only by controlling genes required for CC progression but also by regulating genes relevant for fate control and linage specification.

## Control of Fate Decisions in G_1_

**FIGURE 3:**
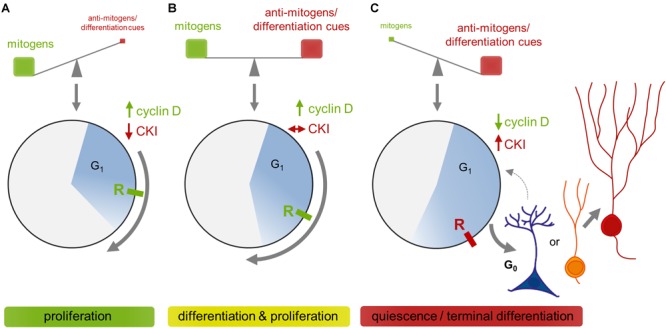
Simplified model on the coordination of *G_1_* dynamics by extrinsic signals and their integration into fate decisions based on evidence from cyclin and CDK manipulation studies. **(A)** Fast proliferation is achieved in presence of high mitogen levels that stimulate the expression of D-cyclins, thus initiating the canonical CC cascade driving progression through *G_1_*. If cells pass the R-point (point of no return), mitogen stimulation is no longer required for commencing cell division. D-cyclin levels stay high until mitogens are withdrawn. **(B)** If specification signals come on board, CKIs increase on top and restrain but don’t block the activities of D-cyclins, resulting in a lengthening of *G_1_*. This, as proposed by the “cell cycle length theory,” provides sufficient time for specification signals to initiate the differentiation program before cells reach the R-point. **(C)** If mitogens decrease, D-cyclins become rapidly degraded and cells can not overcome the R-point. Adult NSCs then return into a reversible quiescent state (G_0_), whereas cells that initiated their differentiation program terminally exit the cell cycle to become an immature neuron (IN).

The commitment of cells to divide requires the presence of external mitogenic signals. It is during early G_1_ that cells are highly susceptible to such extrinsic cues and make critical decisions about their fate, including the commitment to proceed, pause or exit the CC (Matsushime et al., 1994; Sherr, 1994a; Pauklin and Vallier, 2013). In the presence of mitogens, D-cyclins become upregulated and initiate the cascade driving the cell through the restriction point to commence a new round of division. If mitogens are withdrawn or anti-mitogenic cues are present (e.g., BMPs), D-cyclins are lost and Cip/Kip proteins accumulate, forcing the cell into a reversible quiescent state, a hallmark of aNSCs (G_0_; Blomen and Boonstra, 2007; Andreu et al., 2015). Recent evidence suggests that aNSC quiescence is not merely an inactive state but rather is actively maintained (Wang et al., 2011; Morizur et al., 2018), at least partly by Cip/Kip proteins, whose deletion inevitably leads to excessive proliferation and premature depletion of aNSCs (Kippin et al., 2005; Cheung and Rando, 2013; Andreu et al., 2015). Exposure to differentiation cues is also accompanied by an increase in Cip/Kip CKIs, but this time forcing differentiation and terminal exit from CC (Lukaszewicz et al., 2002; Blomen and Boonstra, 2007; Horster et al., 2017). Of note, differentiation and proliferation are not mutually exclusive events, as is the case in neuronally committed progenitor cells. Cells rather continue to divide at slower pace and cease cycling toward the end of the differentiation process (Lukaszewicz et al., 2002; Coffman, 2003). Such a lengthening of the CC during neuronal differentiation has been initially described for the embryonic cortex (Takahashi et al., 1995). Subsequent studies applying sophisticated genetic tools to selectively manipulate important G_1_ regulators suggest that the length of G_1_ can directly influence proliferation/differentiation decisions in NPCs (Figure 3). For example, overexpression of cyclin D1-CDK4 complexes, or cyclin D1 and cyclin E alone were found to shorten G_1_, which was accompanied by a deceleration of neurogenesis and enhanced expansion of IPCs, both in embryonic and adult neurogenic niches (Lange et al., 2009; Pilaz et al., 2009; Artegiani et al., 2011; Bragado Alonso et al., 2019). Conversely, silencing of cyclin D-CDK4, deletion of CDK6 or pharmacological inhibition of CDK2 or CDK4 lengthen G_1_ and lead to premature neuronal differentiation (Calegari and Huttner, 2003; Lange et al., 2009; Beukelaers et al., 2011; Lim and Kaldis, 2012; Roccio et al., 2013). These observations have led to the hypothesis that CC lengthening may be a cause of differentiation, proposing that a longer G_1_ will increase the propensity of fate-determining signals to accumulate and drive differentiation (Calegari and Huttner, 2003; Salomoni and Calegari, 2010). However, for the adult DG there is also evidence not completely in line with this hypothesis, coming from two studies which examine the correlation between voluntary exercise-induced proliferation and CC length (Farioli-Vecchioli et al., 2014; Fischer et al., 2014). Whereas the study of Fischer et al. (2014) despite finding a tendency toward G_1_ shortening, reports no clear correlation between CC length and increased proliferation after a short period (5 days) of running, Farioli-Vecchioli et al. (2014) showed that the proliferative response after prolonged (12 days) running is accompanied by shortening of the CC, which was attributable to shortening of the S phase in neuronally committed progenitors. This is in line with others reporting that NPCs shorten S phase upon neuronal commitment both during development and in the adult DG (Arai et al., 2011; Brandt et al., 2012). Interestingly, Brandt and colleagues further observed that radial aNSCs, once activated, divide faster, also through shortening of their S phase, which is in agreement with the “disposable stem cell” model as put forward by Encinas et al. (2011), but in contrast to the “long-term repeated aNSC self-renewal” model proposed by Bonaguidi et al. (2011) and to the finding that expanding NPCs spend more time in S phase for DNA quality control (Arai et al., 2011). Taken together, the overall picture is more complex than previously assumed, and it still remains to be proven whether and how changes in CC dynamics translate into fate decisions. A possible explanation for the apparent discrepancies described before is that the G_1_ proteins manipulated to induce changes in CC length may in addition to their CC regulatory function directly influence cell fates. Indeed, several studies have provided evidence for direct roles of G_1_ proteins in differentiation, independent of their CC regulatory activity, through interaction with signaling pathways and transcription factors controlling fate decisions (Pauklin and Vallier, 2013; Pauklin et al., 2016). For cyclin D1, chromatin-wide location analyses identified hundreds of target genes, including *Notch1* and *Wnt3*, suggesting direct roles in the maintenance and differentiation of stem cells (Bienvenu et al., 2010; Pauklin et al., 2016). P27^Kip1^ has been shown to promote differentiation through transcriptional repression of *Sox2* and stabilization of the proneural protein Ngn2 (Vernon et al., 2003; Nguyen et al., 2006; Li et al., 2012). Ngn2 is moreover targeted by CDKs through direct phosphorylation at multiple sites (Ali et al., 2011). This results in quantitative inhibition of Ngn2’s ability to induce transcription of differentiation genes, thus providing a mechanism how CDK activity and perhaps also CC length can be sensed to coordinate neuronal differentiation.

## Concluding Remarks

Recently there has been a remarkable progress in our understanding about the control of adult hippocampal neurogenesis by the CC. However, despite accumulating evidence for multiple and diverse interactions between CC components and fate decisions of NPCs, we are far from drawing a comprehensive picture. Still, several CC proteins await investigation in adult NPCs or are subject to ongoing work. On the other hand, interpretation of existing data is frequently complicated because many G_1_ regulators, including D-cyclins and CDKs, (i) emerge as pleiotropic molecules regulating multiple cellular functions, often independent from their CC regulatory role and localized to different protein domains, and (ii) display functional redundancy and counter-regulate if the expression of related proteins is experimentally manipulated. Additional detailed analyses of expression patterns in combination with spatiotemporally controlled manipulation of genes, as well as of individual functional domains of CC regulators, may help to answer outstanding questions. We believe that a detailed understanding of the regulatory mechanisms underlying adult neurogenesis may open new avenues for developing therapies of neurodegenerative diseases. The first attempts to exploit this potential have already been made and have produced promising results.

## Author Contributions

AU conceptualized and wrote the manuscript and prepared the figures. OW conceptualized and edited the manuscript.

## Conflict of Interest Statement

The authors declare that the research was conducted in the absence of any commercial or financial relationships that could be construed as a potential conflict of interest.

## Abbreviations

aNSC: adult neural stem cells
CC: cell cycle
DG: dentate gyrus
IPC: intermediate or transit-amplifying progenitors
NPC: neural precursor cells (undistinguished pool comprising NSC, IPC, and neuroblasts)
NSC: neural stem cells
SGZ: subgranular zone
SVZ: subventricular zone
VZ: ventricular zone
